# Cleansing Milk

**Published:** 1920-03-20

**Authors:** 


### Cleansing Milk.

We do not drink enough milk. That is agreed by all who can speak with authority. To young children it is of vital importance. Concurrently with the advocacy of this incontestable point of view—it should, in fact, precede advocacy were that possible—there is needed a comprehensive effort to cleanse the milk supply. One of the great difficulties in improving the supply of milk in the past has been pointed out by the political correspondent of the *Manchester Guardian,* who says it has been due to the fallacious theory that milk was only of one quality. Consequently you had clean milk and dirty milk, raw milk and pasteurised milk, all sold under the general name of milk. The man who went to the expense of producing milk that was cleaner and safer than the ordinary milk had no means of getting a return by means of higher price for the additional care and expense in preparing this higher-quality article. Similarly there was nothing to prevent the producer or retailer of bad-quality milk from calling it "nursery milk," "children's milk," or "pure milk," and selling it at a higher rate than the market price, if by his advertisements he was able to persuade people to pay this higher price. A large amount of the milk that was sold as raw milk had in fact at some period been artificially treated by heat, not in order to make it an absolutely safe article, as would be done under efficient pasteurisation, but in order to make it keep longer. This artificial treatment, in fact, was in the interests of the seller and not of the consumer.

It might fairly be claimed by the Government that they are giving a lead on this subject. They intend shortly to introduce a measure amending, in the light of later knowledge, the Milk and Dairies Act of 1915, which has not yet been put into operation. This will represent a considerable effort to cleanse the supply, to define and enforce strictly different grades of milk, and, by licensing producers and dealers, give authoritative supervision. It is good to find a statement by Lord Astor, a member of the Government, that, before anything else is done, there must be careful inspection of all farms to find out the conditions of production. That is beginning at the beginning.

During the war Lord Astor's committee, which had control of the milk supply, issued a scale of the requirements of infants and children according to age groups, in order to enable local authorities to decide upon the priority of claims of different groups of milk drinkers. "If" (says Lord Astor in the *Manchester Guardian*) "all infants and children got the amount of milk which it was laid down they ought to get, there would be no milk for adults for nearly three months in the winter. We know that almost every adult drinks some milk, and consequently the total supply available for and drunk by children is far below their requirements. A census was taken by the Ministry of Food in January 1918. The returns showed that the average consumption of milk per head per day for Great Britain was a quarter of a pint, varying from one-tenth of a pint in Inverness to one-third of a pint in London. This is less than half the ordinary

**THE PUBLIC WELL-BEING**—*(continued)*

consumption in the city of New York, and it is far below the needs of children.

"It is most important that people should realise the extent to which in the national interest we should develop the dairy industry," he went on. "Incidentally my Committee recommended the extension of goat-keeping, particularly for scattered rural areas. Goats can support themselves on the roughest grass, are easily kept, and are quite hardy."

Even at present prices milk is the cheapest form of food per calory.

"There is one factor," said Lord Astor, "that is going to affect milk production. Before the war dairying was the safest branch of farming—that is, it was the least subject to violent alternations of boom and depression. It is because of this that a large number of farmers produced milk. Now, however, the Corn Production Act has given the wheat-grower a greater feeling of security. Consequently milk producers are tending to ask for a larger profit. It has therefore become largely a matter of educating the farmer in scientific methods of increasing the yield. If the yield of every cow were increased by one-tenth of a gallon per day the cost of milk would automatically drop 2d. per gallon without reducing the profits of the farmer. An increasing number of farmers are realising that the way to make a profit is not by struggling to put up prices, but rather by reducing the cost of production."

